# Ecosystem stability relies on diversity difference between trophic levels

**DOI:** 10.1073/pnas.2416740121

**Published:** 2024-12-06

**Authors:** Yizhou Liu, Jiliang Hu, Jeff Gore

**Affiliations:** ^a^Physics of Living Systems, Department of Physics, Massachusetts Institute of Technology, Cambridge, MA 02139; ^b^Department of Mechanical Engineering, Massachusetts Institute of Technology, Cambridge, MA 02139

**Keywords:** ecology, stability, diversity, trophic level

## Abstract

The stability of ecological communities has a profound impact on humans, ranging from individual health influenced by the microbiome to ecosystem services provided by fisheries. A long-standing goal of ecology is the elucidation of the interplay between biodiversity and ecosystem stability, with some ecologists warning of instability due to loss of species diversity while others arguing that greater diversity will instead lead to instability. Here, by considering a minimal two-level ecosystem with multiple predator and prey species, we show that stability does not depend on absolute diversity but rather on diversity differences between levels. We found that increasing diversity in either level first destabilizes but then stabilizes the community (i.e., a reentrant stability transition). We therefore find that it is the diversity difference between levels that is the key to stability, with the least stable communities having similar diversities in different levels. An analytical stability criterion is derived, demonstrating quantitatively that the critical diversity difference is determined by the correlation between how one level affects another and how it is affected in turn. Our stability criterion also applies to consumer–resource models with other forms of interaction such as cross-feeding. Finally, we show that stability depends on diversity differences in ecosystems with three trophic levels. Our finding of a nonmonotonic dependence of stability on diversity provides a natural explanation for the variety of diversity-stability relationships reported in the literature, and emphasizes the significance of level structure in predicting complex community behaviors.

## Significance

Understanding ecosystem stability and its relationship to biodiversity is critical for ensuring the sustainable development of societies. This research challenges the conventional focus on absolute species diversity, revealing that stability in ecosystems depends on the difference in diversity between trophic levels. We found that ecosystems with similar diversities across different trophic levels are least stable, whereas a greater difference in diversity across trophic levels stabilizes the ecosystem, regardless of which level has greater diversity. These findings offer a perspective on the diversity-stability debate and provide a quantitative criterion for predicting ecosystem stability across various interaction types, enhancing our ability to forecast and manage ecosystem responses to biodiversity changes.

Natural communities, from a microbiome to a forest, have a profound impact on humanity, influencing individual well-being and the sustainable development of societies (1, 2). Within these ecosystems, the interplay among millions of organisms from numerous species both within and between trophic levels (3, 4)—such as fighting for territories (5), predation (6–8), competing for resources (9–11), and cross-feeding (12–15)—creates a wide range of dynamic behaviors. These behaviors span a spectrum from global stability (16–18) to rich, complex dynamics that include multistability (19–24), periodic oscillations (6–8), and even chaotic fluctuations (25–27). Recognizing the interdependence of biodiversity and ecosystem stability, ecologists have spent decades striving to decode the mechanisms that enable complex ecosystems to achieve and sustain robust stability and functioning. Their work seeks to illuminate how these myriad interactions contribute to the resilience of natural communities, which in turn impacts the stability of the environments we depend on.

Since Robert May’s pioneering work 50 y ago (28), theoretical ecologists have used interspecies interaction strength and biodiversity to identify instabilities of large communities. May’s result predicts that communities will be destabilized by either strong interactions between species or a large number of species (28). Subsequent research, employing generalized Lotka–Volterra (gLV) models, delved deeper into ecological networks without specifying mechanisms of interactions (29–31), examining how interaction patterns, such as the proportion of mutualistic versus competitive relationships (29, 30), impact the stability of ecosystems. One of the main takeaways was that increasing diversity is still expected to destabilize ecosystems, a robust theoretical expectation that was potentially at odds with empirical results that diversity can either stabilize or destabilize communities (32–40). Recent investigations have revealed that higher-order interactions (41, 42), constraints on interactions (31, 43), or self-regulation (44, 45) can cause increased diversity to stabilize ecosystems, which may help to understand empirical patterns. A major question is to understand the structure of interactions within an ecosystem and how it will alter the theoretically predicted diversity-stability relationship.

In particular, predation or resource competition and the corresponding trophic level structures are important in real-world communities, whose impact to diversity-stability relationships is relatively poorly understood (3, 46). Predators may have only limited direct interactions but may instead compete primarily through competition for prey at a lower trophic level. More generally, a food web can include abiotic resources as a level in addition to trophic levels, where, for example, bacteria near the bottom of a food web may primarily compete for abiotic resources such as sugars. In considering the specific mechanisms like resource competition (47–57), predation (58–61), cross-feeding (12, 47, 52, 55), pH (27, 62), etc., the models incorporate key aspects of natural ecosystems but in many cases cannot be analyzed analytically. A variety of models have suggested that cross-feeding (50, 52, 55), variation in yields (48, 54, 63–65) (how efficient prey/resources are converted to predator/consumer biomass), and toxicity (47) can destabilize communities, while some mechanisms like predator interference (59) can stabilize communities. Stability criteria based on deterministic matrices successfully predict the existence of instability due to these various mechanisms (50, 52, 55). However, we do not have an explicit stability criterion based on simple community statistics, leaving the relationship between diversity and stability in the presence of trophic structure unclear.

Here, we focus on a minimal ecosystem model with two levels that could describe either predator–prey or consumer–resource interactions. In the context of predator–prey interactions these would typically be considered two trophic levels, whereas for consumer species growing on abiotic resources these would be the two lowest levels of a food web. We found a reentrant stability transition in which increasing the species diversity in either level first destabilizes but then later stabilizes the fixed points, a phenomenon which is absent in models without trophic levels. We then analytically derive a general stability criterion that quantitatively agrees with numerical results, revealing that stability prevails when the diversity difference between levels is substantial, irrespective of total diversity of the ecosystem. The critical diversity difference is determined by the correlation between how one level affects another and how it is affected in turn. We also find that mechanisms beyond predation or resource competition such as cross-feeding (production of resources by consumers) can destabilize the community, yet the stability criterion that we derived nonetheless correctly predicts the stability boundary. Beyond two-level ecosystems, numerical results further show that diversity difference remains the key determinant of stability in three-level ecosystems, suggesting that diversity differences between levels may play an important role in many ecosystems. Our finding of a nonmonotonic dependence of stability on diversity provides a natural explanation for the variety of diversity-stability relationships reported in the literature. Our work emphasizes the significance of level structure in predicting community behaviors, and provides insights into ecosystem stability.

## Results

To study the stability of an ecosystem with two levels we consider a simple model of predators (or consumers) in the higher level that grow based on consumption of the prey (or resources) in the lower level. We employ the dynamics for the predator abundance $\left(S_{i}\right)$ and prey abundance $\left(R_{\alpha }\right)$ given by (47, 49, 52, 56, 59):

[1] $$\frac{dS_{i}}{dt}=S_{i}\left(\sum_{\alpha }C_{i\alpha }Y_{i\alpha }R_{\alpha }-\delta_{i}-\epsilon_{i}S_{i}\right),$$

[2] $$\frac{dR_{\alpha }}{dt}=h_{\alpha }\left(R_{\alpha }\right)-R_{\alpha }\sum_{i}C_{i\alpha }S_{i}.$$

**Fig. 1.**
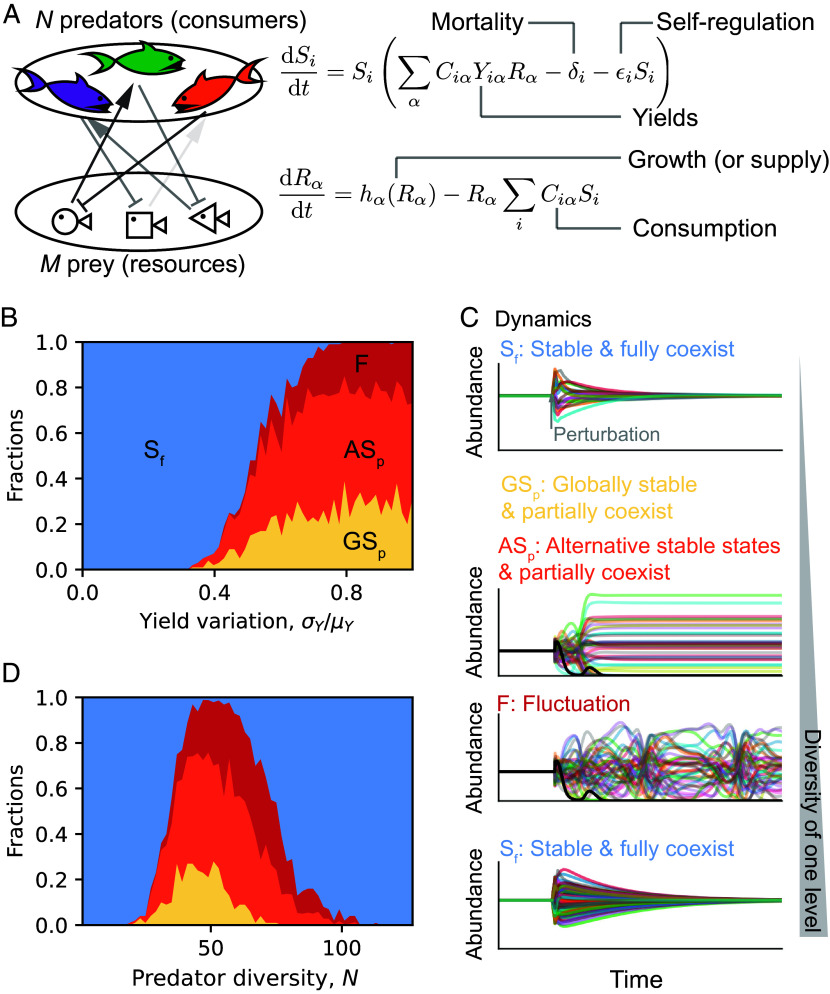
Two-level ecosystems that could describe either predator–prey or consumer–resource dynamics are first destabilized but then stabilized by increasing predator (or prey) diversity at the fixed point. (*A*) A minimum model with N predator species and M prey species. Consumption ($C_{i\alpha }$) depletes prey while promotes predator growth. Prey consumed transfer to predator biomass increment subject to yields ($Y_{i\alpha }$). Prey growth (or resource supply) is governed by $h_{\alpha }$ function. Predator death is encoded in mortality rates ($\delta_{i}$). And predators may have self-regulation ($\epsilon_{i}$) limiting growth. (*B*) Only changing the variation of yields, we observe one transition to instability with multiple possible behaviors after the loss of stability of the original fixed point (S_f_): the community can be globally stable but with extinction (GS_p_), or it can go to alternative stable states with partial coexistence, or it can display persistent fluctuations (F). We fixed prey diversity M=64 and predator diversity N=32 (details in *SI Appendix*). (*C*) Time series examples for different dynamical behaviors. We plot the abundances normalized by their original fixed-point values. At first, the communities are at the given fixed point. Later, communities are perturbed (starting from another randomly sampled initial condition). If the time series come back to the given fixed-point values, the community is S_f_. GS_p_ and AS_p_ share one illustrative time series as they all end up being stable but having extinctions (highlighted by the dark curve). (*D*) Only varying predator diversity, N, the fraction of S_f_ first decreases but then increases with N (reentrant stability transition). The color encoding for different dynamical behaviors is the same as (*B*). We fixed prey diversity M=48 and varied N from 1 to 128, and the fractions of different dynamical behaviors in the figure were averaged over communities with the same N (details in *SI Appendix*).

For concreteness, one can imagine the abundances are measured by number of individuals or mass per area, which does not affect our results. Here, $h_{\alpha }\left(R_{\alpha }\right)$ refers to how prey grow, i.e., logistic growth $h_{\alpha }\left(R_{\alpha }\right)=g_{\alpha }R_{\alpha }\left(K_{\alpha }-R_{\alpha }\right)$, or how resources are supplied to the system, i.e., chemostat supply $h_{\alpha }\left(R_{\alpha }\right)=l_{\alpha }\left(\kappa_{\alpha }-R_{\alpha }\right)$. Our conclusions are valid for either functional form (*Methods*) and therefore we do not emphasize the function $h_{\alpha }$ in the text. Numerical results in the main text are obtained from logistic growth while we present the counterparts for chemostat in *SI Appendix*, Fig. S1. The above equations describe only predation or resource competition (Fig. 1*A*), but we can generalize it to include cross-feeding and resource–resource regulation under the consumer–resource picture (*Methods*). $C_{i\alpha }$ describes the per capita consumption rate for predator (species) i on unit prey (species) α. Prey consumed will not be fully converted to biomass of predator, so $Y_{i\alpha }$ quantifies the associated transfer efficiency or yield (yield $Y_{i\alpha }$ can be negative capturing toxicity). We aim to study the stability of the ecosystem at a fixed point, and the linear consumption and growth can then be thought of as local linear approximations of some complicated consumption and growth functions. Without loss of generality, we assume the fixed point of interest has N predators and M prey and focus on their dynamics. Finally, predators are set to have mortality $\delta_{i}$ and self-regulation $\epsilon_{i}$. In the canonical consumer–resource models, there is no self-regulation for consumers ($\epsilon_{i}=0$) (49, 50, 52, 55), while in predator–prey models, predator self-regulation is common (59–61). We include self-regulation for generality, which also overcomes the competitive exclusion principle (66–68) (i.e., the number of predators N that can coexist being limited by the number of prey M). We initially assume predator self-regulation is much smaller than other interactions for analyticity but later discuss how predator self-regulation will shift the analytic results.

In a large community we expect only the statistical properties of parameters to determine the stability. Since the growth rate of a predator cannot go to infinity when there are more available prey, individual consumption rates $C_{i\alpha }$ should scale as 1/M. We therefore sample $C_{i\alpha }$ with mean and SD denoted by $\mu_{C}/M$ and $\sigma_{C}/M$, respectively (the actual distribution does not influence the results, main text figures are obtained with $C_{i\alpha }$ uniformly in [0,1/M]). The yields are determined by biochemical processes and should not scale with community size. We sample yields, $Y_{i\alpha }$, from a Gaussian with mean, $\mu_{Y}$, and SD, $\sigma_{Y}$ (see details in *Methods*). If not specified, we will use μ and σ to denote means and SD of other parameters, with proper subscript showing which parameter we are referring to.

Although the original MacArthur (consumer–resource) model only displays global stability (56), allowing yields to vary depending on both predators and prey can lead to the loss of stability (48, 54, 63, 64). Predators (consumers) may digest prey (resources) differently and prey (resources) can contain nutrients differently, which leads to yield variation observed (48). One way to understand why variation in yields destabilizes the community is that it can lead to “niche encroachment,” whereby predators consume prey important for the growth of other predators (54). Indeed, sampling yields as i.i.d. and increasing yield variation, $\sigma_{Y}/\mu_{Y}$, causes a loss of stability and a variety of community outcomes (Fig. 1*B*, numerical details in *SI Appendix*). Some communities remain globally stable but with some extinction, whereas other communities display sustained fluctuations in abundance (limit cycles and chaos) or alternative stable states with different community compositions (Fig. 1*C*). After the original fixed point becomes unstable, the final diversity was shown to depend on the degree of instability or encroachment (54). Based on the idea of niche encroachment, we would expect that, holding the variation in yields constant, increasing the number of predators will make the niches more crowded and therefore lead to instability.

To test this expectation, we studied how diversity of the top level (number of predator species, N) affects community stability. We fixed other statistical parameters (where $\sigma_{Y}\neq 0$) and prey diversity (number of prey species, M) when sampling communities but varied predator diversity, N, and then simulated the dynamics of each sampled community (details in *SI Appendix*). With a small number of predators, all communities are globally stable at the original fixed point where all N predators survive (Fig. 1*D*). As expected, increasing predator diversity can lead to the loss of stability of the original fixed point and the full range of rich dynamical behaviors discussed previously. Although increasing the number of predators in the top level led to a loss in stability, we were surprised to find that further increases in predator diversity led the community to regain stability (Fig. 1*B*). The observation that stability depends nonmonotonically on predator diversity contradicts the idea that more predator species leads to more niche encroachment and only destabilize communities. We therefore observed a counterintuitive reentrant stability transition with respect to predator diversity, i.e., increasing predator diversity first destabilizes but then stabilizes the communities.

Having characterized community outcomes after varying the diversity of the top level (number of predator species), we next sought to characterize community outcomes when varying the diversity of the lower level (number of prey species). We therefore sampled communities only varying prey diversity, M, and again found a reentrant stability transition in which communities lost but then regained stability (*SI Appendix*, Fig. S2). Our observation that stability depends nonmonotonically on prey diversity also contradicts the idea that more prey species provide more niches and only stabilize the fixed points.

**Fig. 2.**
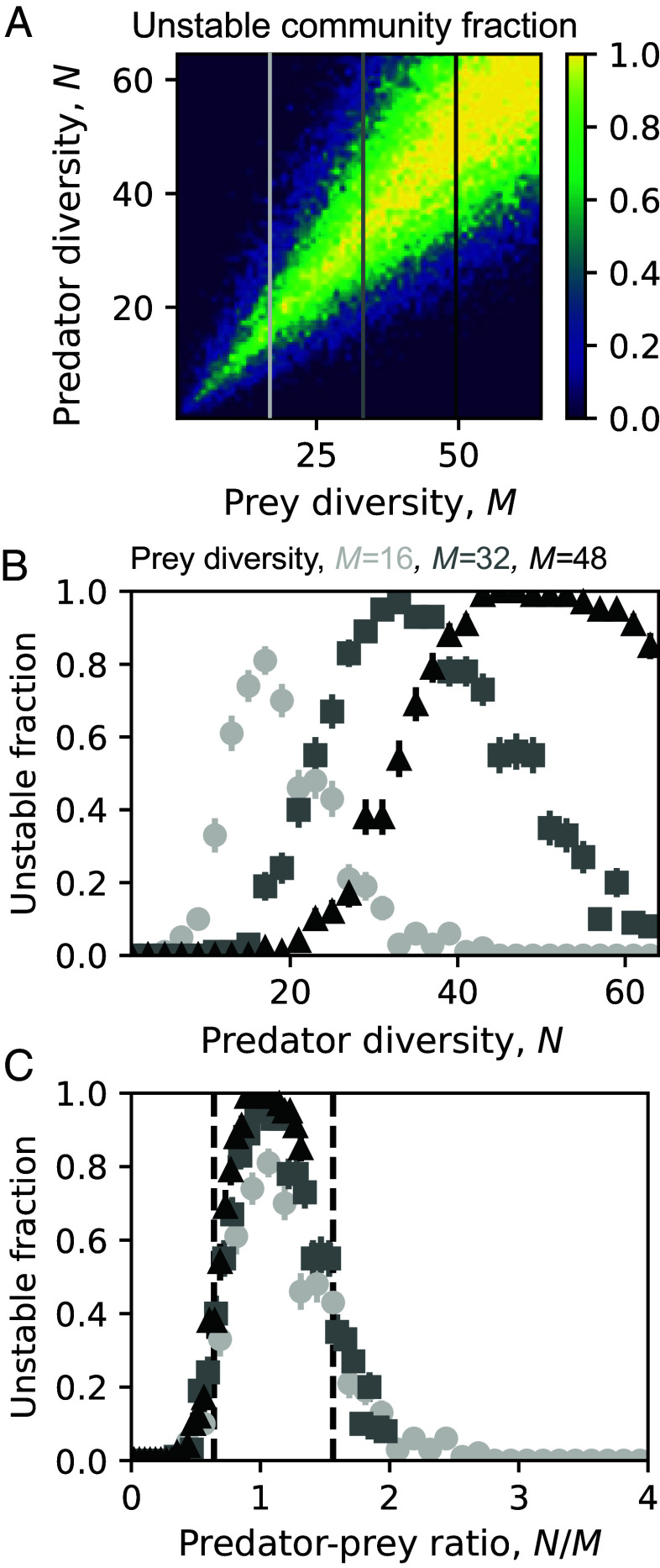
Stability depends on diversity difference between trophic levels, with a greater difference leading to stability. (*A*) For communities with different number of prey, stability depends differently on predator diversity, but the fraction of unstable communities seems to have a peak near N=M, indicating the universal importance of N/M. The color gradient represents the fraction of unstable communities, with higher values indicating a greater fraction of instability. The vertical lines indicate different levels of prey diversity (M=16,32,48). (*B*) Fraction of unstable communities is plotted in more detail for selected prey diversity (M=16,32,48), which shows different dependence on predator diversity. (*C*) There is indeed a universal trend of stability for different communities if we vary the ratio of number of predators to that of prey, N/M: communities will first be destabilized but then stabilized (reentrant stability transition). (*B* and *C*) used the same set of data. Each data point in the figure was averaged over 100 sampled communities. Error bars represent SEM.

To gain insight into the reentrant stability transition that we observed, we next varied predator diversity N and prey diversity M while holding other statistics unchanged. We observed that the fraction of unstable communities depends differently on predator diversity N for different prey diversity M (Fig. 2 *A* and *B*). Nevertheless, instability depends on N and M in a similar way, with the most unstable cases being when the diversities of the two levels are similar (N≈M). Surprisingly, after we normalize the predator diversity by prey diversity, we found a universal pattern that increasing predator–prey ratio, N/M, first destabilizes but then stabilizes communities, giving two stability-instability boundaries (Fig. 2*C*). The stability of our two-level community therefore depends fundamentally on the difference in diversities of the two levels, with the community being least stable when the diversities in the two levels are similar.

**Fig. 3.**
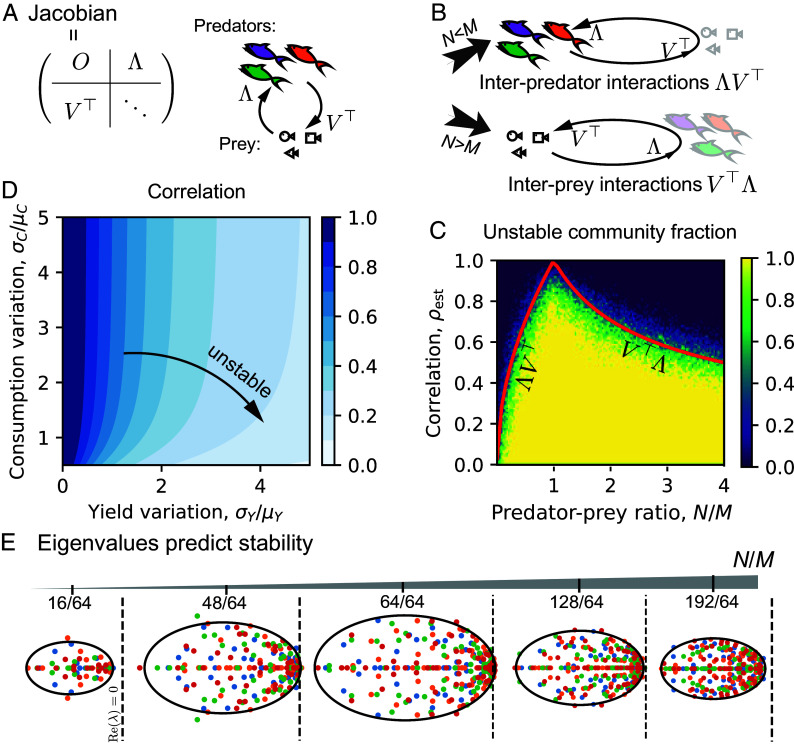
Stability relies on the predator–prey ratio and the correlation between how predators affect prey and how prey in turn influence predator. (*A*) The local Jacobian at the fixed point with negligible predator self-regulation is a block matrix mainly consisting of how prey affect predators (the matrix Λ), how predators affect prey (the matrix $V^{T}$), and effective prey self-regulation (lower right diagonal matrix). (*B*) When there are more prey species than predator (N<M), stability depends on the matrix $\Lambda V^{T}$ (reduced interpredator interactions) and effective self-regulation, and will be determined only by the stability of $\Lambda V^{T}$ in large communities. When there are more predators than prey (N>M), similarly, stability for large communities will be determined by that of the matrix $V^{T}\Lambda$ (reduced interprey interactions). (*C*) In different regimes (N<M and >M), the stability boundaries are derived (red lines), depending on predator–prey ratio and the correlation between Λ and -V elements (estimated here from mechanistic parameters), agreeing well with numerical results. The heatmap is obtained from numerically sampled communities with M=32 and N varying from 1 to 128. There are 128×100 pixels, and each pixel has 10 communities, from which the unstable community fraction can be calculated. (*D*) The estimated correlation (Eq. **6**), which is exact when yields and consumption are i.i.d., respectively) depends on the variation in yields, $\sigma_{Y}/\mu_{Y}$, and the variation in consumption, $\sigma_{C}/\mu_{C}$. (*E*) The eigenvalues of $\Lambda V^{T}$ (or $V^{T}\Lambda$ when N/M>1) are in an ellipse in the complex plane, and stability depends on if the ellipse goes beyond the dashed line (the dashed lines mark where the real part is zero; note the real axis is horizontal and imaginary axis is vertical by convention).

We next sought to explain the surprising reentrant stability transition that we observed with respect to predator–prey ratio. We followed the standard stability analysis to study the Jacobian at the fixed point (Fig. 3*A*). If we combine predator and prey abundances into one vector, the Jacobian becomes a block matrix, where in our convention the upper right part, Λ (an N×M matrix), encodes how prey affect predators, the lower left part, $V^{T}$ (an M×N matrix), encodes how predators modify prey, and the lower right part is an M×M diagonal matrix encoding effective self-regulation of prey (*Methods*). In the regime N<M, with the proper approach of calculating block matrix determinant, we can have M-N stable eigenvalues directly from prey self-regulation, and solve the rest of 2N eigenvalues from eigenvalues of $\Lambda V^{T}$ via quadratic equations (*Methods*). Since $\Lambda V^{T}$ is an N×N matrix passing the effects from predator-changed prey back to predators (Fig. 3*B*), it is naturally understood as (reduced) interpredator interactions. In large complex systems, we can show that whether the Jacobian can have unstable eigenvalues depends on whether $\Lambda V^{T}$ has unstable eigenvalues (*Methods*). In other words, stability of the large community is determined by reduced interpredator interactions when N<M. By symmetry, one can imagine that in the regime N>M, stability is determined by reduced interprey interactions, $V^{T}\Lambda$ (Fig. 3*B*), which we can prove (*Methods*). Symmetry or duality therefore suggests that there will be two instability transitions, with the transition at N<M determined by interpredator interactions ($\Lambda V^{T}$) and the transition at N>M determined by interprey interactions ($V^{T}\Lambda$).

After simplifying the stability analysis to studying interpredator or interprey interactions, we then could derive an analytic and complete stability criterion. For large communities, $\Lambda V^{T}$ (or $V^{T}\Lambda$) can be viewed as a large random matrix and we can apply the non-Hermitian Marchenko–Pastur law (54, 69, 70) (*Methods*). The eigenvalues of $\Lambda V^{T}$ are then distributed in an ellipse in the complex plane (up to a nonessential normalization not changing signs of eigenvalues). The ellipse has a center on the real axis at -ρ(1+N/M), whose major axis is also on the real axis and has length $\sqrt{N/M}\left(1+\rho^{2}\right)$, where -ρ is the correlation between $\Lambda_{i\alpha }$ and $V_{i\alpha }$ (Fig. 3*E*). Stability requires that all eigenvalues have negative real part, leading to the stability criterion when N<M given by

[3] $$\rho \geq \sqrt{N/M}.$$

For $V^{T}\Lambda$, we only need to exchange N and M in the derivations related to $\Lambda V^{T}$ and can directly obtain the criterion for N>>M given by

[4] $$\rho \geq \sqrt{M/N}.$$

[5] $$\rho \geq \sqrt{\text{min}\left(N/M,M/N\right)}.$$

We can combine the stability criteria in different regimes into one universal criterion as Random matrix analysis therefore allows for a simple stability criterion that depends upon how the ratio of diversities in the two levels compared to the correlation in interactions between the levels.

We next explored the meaning of the interlevel correlation ρ, and how it is affected by mechanistic parameters connecting the predators and prey. As a general statement, the correlation ρ measures the alignment between how predators affect prey and how prey in turn influence predators. This statistical parameter quantifies the degree of reciprocity or feedback between these two levels. For example, if a predator species kills certain prey at high rates but cannot grow well on that prey, the correlation ρ is reduced. On the other hand, if all yields are uniform and positive then the correlation is maximized as 1. We estimated the correlation when yields have variation as (see *Methods* for derivation)

[6] $$\rho_{\mathrm{est}}=\sqrt{\frac{1}{1+\frac{\sigma_{Y}^{2}}{\mu_{Y}^{2}}+\frac{\sigma_{Y}^{2}\mu_{C}^{2}}{\sigma_{C}^{2}\mu_{Y}^{2}}.}}$$

With this estimated correlation, we have a stability criterion that purely depends on mechanistic parameter properties and diversities, which is well supported by numerical experiments (Fig. 3*C*, where the red line is theoretical stability boundary and the heatmap is numerical results on stability). For a community with given sizes (N and M), greater variation in yields, quantified by $\sigma_{Y}/\mu_{Y}$, or smaller variation in consumption, $\sigma_{C}/\mu_{C}$, can reduce the correlation between predators affecting prey and prey impacting predators, tending to destabilize the community (Fig. 3*D*). A greater yield variation makes growth rates more different from consumption rate, i.e., predators more likely to encroach upon niches (growth promoting prey) of others, and thus tend to result in instability (54). Similarly, small consumption variation means predators are competing for a very similar set of prey, leading to strong interactions and subsequently, instability. We have therefore rationalized reentrant stability transitions via the symmetric roles of interpredator and interprey interactions, identified a critical diversity difference for instability controlled by correlation, and interpreted this correlation as the alignment between interlevel interactions or how well species focus on their own niches.

**Fig. 4.**
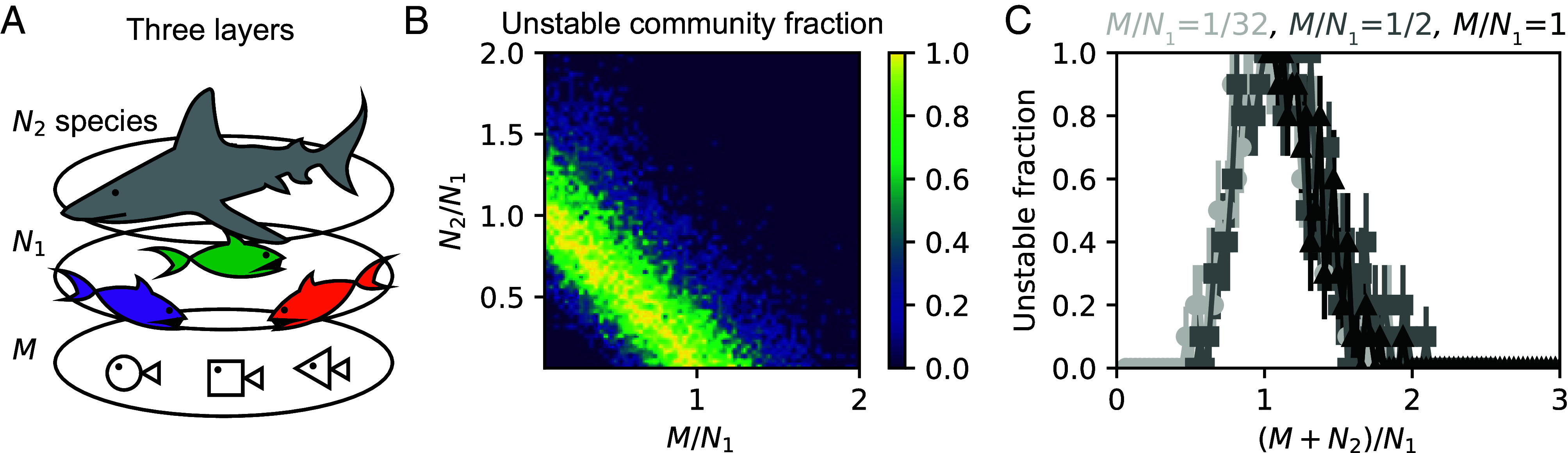
Numerical evidence suggests that stability also depends on diversity differences in communities with three levels. (*A*) Illustration of a three-level community has M species in the bottom level, $N_{1}$ species in the middle level, and $N_{2}$ species in the top level. (*B*) For one instance, we found stability depends nontrivially on both ratios of diversities (of nearby levels), $M/N_{1}$ and $N_{2}/N_{1}$, with the most unstable cases having $M+N_{2}\approx N_{1}$. We kept $N_{1}=32$, while changed M and $N_{2}$ from 1 to 64, respectively. (*C*) The diversity difference quantified by $\left(M+N_{2}\right)/N_{1}$ seems to determine stability transitions, as different reentrant transitions collapse if we plot the fraction of unstable communities with respect to $\left(M+N_{2}\right)/N_{1}$. Data in (*C*) are from (*B*). Error bars represent SEM. We assumed for the instance shown that there is no direct interaction between top and bottom levels, interlevel correlations are all ρ=0.8, and the middle level has negligible self-regulation as in conventional consumer–resource models. The multilevel communities need some levels to have sufficiently weak self-regulation to be unstable, and the instability seems to depend on difference between diversity of levels having negligible self-regulation and that of other levels (see more discussions and examples in *SI Appendix*).

Beyond cases we could analyze analytically, we further explored the generality of our findings through numerical tests. Natural ecosystems may have more than two levels in the food web, where our results cannot be directly applied. To gain some insight into more complex ecosystems, we studied stability of a three-level community containing *M* prey species, $N_{1}$ predator species on those prey, and $N_{2}$ apex predators that consume the middle predators (Fig. 4*A*). We assumed that there are only direct interactions between neighboring levels and the correlations of interlevel interactions are the same (see details in *SI Appendix*). We found that stability depends on both diversity ratios, $M/N_{1}$ and $N_{2}/N_{1}$, with the most unstable cases having $M+N_{2}\approx N_{1}$ (Fig. 4*B*). This finding inspired us to plot the stability as a function of diversity difference $\left(M+N_{2}\right)/N_{1}$, which then show a unified reentrant transition (Fig. 4*C*). These results suggest that diversity difference between trophic levels may therefore play a special role in determining stability even in multilevel ecosystems.

## Discussion

We have studied a two-level ecosystem (predator vs. prey or consumer vs. resource) where interactions between species are due to predation or competition for food. The level structure leads to the qualitatively new phenomenon of a reentrant stability transition: increasing diversity of either level first destabilizes but then stabilizes the communities. This observation of a return to an original phase (in this case stability) as a function of a control parameter is similar to reentrant phase transitions observed in liquid crystals (71), RNA-polycation mixtures (72), and polypeptides (73) but with very different mechanisms. However, the reentrant phenomenon of “double descent” (74, 75) in machine learning, where more training data first leads to greater errors but then additional data leads to smaller errors, has a similar mathematical origin via random matrix theory, namely the Marchenko–Pastur law (76, 77). These reentrant behaviors are counterintuitive as we do not expect a system to return to its original state when tuning one parameter monotonically, and more work is required to clarify the similarities and differences of the various types of reentrant transitions.

In our communities, predator species in the higher level do not interact directly with each other, but instead all interactions are mediated by competition for the prey (or resources) at the lower level. When there are more prey than predators (M>N), it is the interpredator interactions ($\Lambda V^{T}$) that determine stability. Increasing the number of predators then destabilizes the community, consistent with previous results obtained in fully connected Lotka–Volterra type models (28, 29). In fact, by heuristically comparing our stability criterion to May’s (28), we can obtain an explicit expression for the interaction strength (derived in *Methods*: greater yield variation or fewer prey leads to stronger interaction strength). However, when we have more predators than prey (N>M), it is the interprey interaction ($V^{T}\Lambda )$ that determines stability, meaning that increasing predator diversity stabilizes the communities as it weakens the interprey interactions. The reentrant stability transition is therefore due to this symmetric behavior between predators and prey that emerges in a two-level ecosystem.

The symmetry argument that when N>M we need to shift to consider interprey interactions is one straightforward way to understand the existence of reentrant transitions yet does not present the complete picture. The matrices $\Lambda V^{T}$ and $V^{T}\Lambda$ share the same nonzero eigenvalues, meaning that we can predict stability correctly with interpredator interactions $\Lambda V^{T}$ alone. The reentrant stability transition may therefore arise from interpredator or interprey interactions alone rather than via the transition from interpredator interactions to interpredator ones. A puzzle then emerges as the interpredator interaction $\Lambda V^{T}$ can always predict stability but the intuition for the interpredator interaction, i.e., the niche encroachment, only works for communities with fewer predators than prey. Specifically, why does the intuition that adding predators leads to strong niche encroachment and therefore instabilities work when N<M but fail when N>M? Our naïve interpretation is that when N>M, interpredator interactions are highly correlated (linearly dependent) as predators are competing for fewer prey, which not only leads to zero eigenvalues but also a stabilization effect. Further work is necessary to clarify the ecological intuition behind this stabilization effect that leads to the reentrant stability transition.

The reentrant stability transition can also be observed with a single-level modeling framework in which the two-level structure is contained within the effective interactions. For example, a gLV model containing only the predators but with the interaction matrix given by $\Lambda V^{T}$ will also display the reentrant stability transition with respect to predator diversity (where the number of prey species M is encoded in $\Lambda V^{T}$). The two-level structure of the ecosystem that leads to the reentrant stability transition is in this case encoded in the structure of the interaction matrix of the single-level model of predators.

In our model, we included predator self-regulation whereby increasing abundance of a predator species leads to inhibition of itself. This self-regulation could capture a variety of effects such as competition for territory (5) or intraspecies aggression (78). If predator self-regulation is strictly zero, the number of predators is always smaller than that of prey (N<M) in the absence of fine-tuned metabolic trade-offs (68) and we therefore cannot observe any reentrant stability transition. However, stability still depends on diversity difference between levels (Eq. **3**). We assumed the predator self-regulation is much weaker than other mechanisms and therefore could derive a simple theoretical result for stability (Eq. **5**). If predator self-regulation is nonnegligible, it stabilizes communities, but we can still observe a reentrant stability transition (*SI Appendix*, Fig. S4). Adding strong predator self-regulation therefore stabilizes communities, but stability still depends on diversity difference between levels of a food web.

An important question is the robustness of the reentrant stability transition and associated analysis that we have explained in a simple two-level community. As already shown, the argument that stability depends on diversity differences rather than total diversity still holds for ecosystems with more than two levels, yet the diversity difference can have distinct forms compared to the simple two-level case. Another direction to test robustness is to include more interactions besides predation or resource competition. It is known that cross-feeding under the consumer–resource picture plays an essential role in determining community structure (12–15) and has been show to lead to instabilities (50, 52, 55). Our analysis can include cross-feeding, and we found the stability criterion, Eq. **5**, remains the same while the correlation ρ will be decreased: if one consumer is able to produce metabolites as resources which do not affect its growth rate much, the reciprocity between the two levels is reduced (*Methods* and *SI Appendix*, Figs. S3 and S4). Cross-feeding in our framework therefore destabilizes communities quantitatively as predicted by its effect on ρ. In addition, we found that higher-order dependence of predator growth on prey abundances also destabilizes the communities in a similar manner (*Methods* and *SI Appendix*, Figs. S3 and S4). Our analytic analysis can therefore be generalized to capture the reentrant stability transition that occurs under a range of interactions.

Other modifications to the model may alter the reentrant stability transition in a more profound way. Here, we focused on large ecosystems, where we found that stability is determined by interpredator or interprey interactions. However, in small communities, stability can be lost due to too weak effective self-regulation of prey (*SI Appendix*, Fig. S5). An example is the original Lotka–Volterra predator–prey model (79, 80), where there is only one predator species and one prey species, and the interpredator interaction is always stable ($\Lambda V^{T}$ is a negative scalar number) but the fixed point is not stable. We studied communities lacking interactions within the same level, and the effects of including such intralevel interactions are not clear. Similarly, our preliminary results on three-level communities were obtained from particular examples, and generalizing to include more levels or predation across multiple levels may alter the dynamics (*SI Appendix*, Fig. S6). Future studies are therefore necessary to explore the generality and consequences of the reentrant stability transition identified here.

To connect our theory to observations, we need to know not only the full phase diagram (Fig. 3*C*), but also where natural ecosystems may be located on the diagram. Macroscopic ecosystems may have very inefficient mass transfer leading to restricted yield values. For example, if yields were sampled from uniform distributions between 0 and 0.3 the yield variation is sharply bounded, leading to large interlevel correlation and constraining the ecosystem to the upper region of Fig. 3*C* (*SI Appendix*, Fig. S7). Importantly, a reentrant stability transition is still observed for this constrained sampling of yields. Realistic parameterization therefore suggests which part of the stability criterion is important but does not alter the stability criterion itself.

It has been a long-standing empirical intuition that structures in food webs can alter stability-diversity relationships (40). Greater diversity can enhance stability by providing alternative pathways for energy flow in the food web, thus buffering against the loss of species. However, more species can also lead to instability due to strong interactions. In natural ecosystems, long-term studies in grasslands (18) and meta-analyses of marine ecosystems (81) have generally shown positive diversity-stability relationships. However, these studies often focus on overall diversity rather than the relative diversities across trophic levels. Our theory suggests that it is the ratio of diversities in different levels that is key to stability, providing a possible explanation for the observations of both positive and negative stability-diversity relationships (32–40). Increasing the number of prey species was observed to stabilize communities in some cases (33–35) while destabilizing communities in others (36), which are consistent with our theory. The stabilizing effect of diversity observed in grassland experiments might be due to an increase in plant diversity relative to herbivore diversity. Similarly, in marine ecosystems, the stability of fisheries at higher diversity levels could be related to appropriate diversity ratios between fish species and their prey. The theory may also provide insight into biodiversity patterns across trophic levels. The number of apex predators is usually small (82) and the number of fundamental (abiotic) resources in the bottom of a food web is also limited (83, 84). We therefore can have large diversity differences if middle levels sustain many species, which potentially is one stabilization effect in large natural food webs. However, direct evidence supporting our theory in the literature is lacking, as studies do not typically quantify diversities in different food web levels systematically and record dynamical behaviors simultaneously.

Our findings suggest directions for future empirical research and potential applications in ecosystem management. We propose that future experiments and field studies can vary and measure the diversity ratios between trophic levels, rather than just focusing on overall diversity. Long-term field studies that track changes in diversity across trophic levels and corresponding ecosystem stability would be particularly informative. In terms of applications, our theory could inform conservation strategies by emphasizing the importance of maintaining appropriate diversity ratios across trophic levels, rather than simply maximizing overall diversity. In managed ecosystems such as aquaculture or wastewater treatment systems, deliberately balancing diversities across trophic levels might lead to more stable and resilient systems. For instance, in aquaculture, this might involve carefully managing the diversity of fish species in relation to their feed organisms. However, we caution that real ecosystems are more complex than our model, with factors like spatial structure, environmental variability, and nontrophic interactions that may affect the diversity-stability relationship. Future theoretical work should aim to incorporate these additional complexities to refine our understanding of how diversity differences between trophic levels influence ecosystem stability.

## Methods

We study the dynamics of predator abundances denoted by $S_{i}$ and prey abundances denoted by $R_{\alpha }$. The time evolution is governed by

$$\frac{dS_{i}}{dt}=S_{i}\left(\sum_{\alpha }C_{i\alpha }Y_{i\alpha }R_{\alpha }+\sum_{\alpha ,\beta }H_{i\alpha \beta }R_{\alpha }R_{\beta }-\delta_{i}\right),$$

$$\frac{dR_{\alpha }}{dt}=h_{\alpha }\left(R_{\alpha }\right)+\sum_{i}P_{i\alpha }S_{i}-R_{\alpha }\sum_{i}C_{i\alpha }S_{i}.$$

Say there is an equilibrium of the system with N predators and M prey given by $S^{*}$ and $R^{*}$ (two column vectors). We ignore invasion of other species not at the fixed point.

Besides parameters introduced before, we here add high-order prey regulation for predator growth ($H_{i\alpha \beta }$) and cross-feeding (allowing consumers to produce resource: $P_{i\alpha }$) in terms of consumer–resource picture. There are all kinds of high-order regulations that one prey may help or inhibit the consumption of another prey or itself. For simplicity, we choose $H_{i\alpha \beta }$ to be mean zero (no community level trend for regulations to be beneficial or harmful). And we assume the $H_{i\alpha \beta }$ to have SD $\sigma_{H}/M^{3/2}$ such that if $\sigma_{H}$ is constant, the total effect of regulation summed over all prey/resources has the same order of magnitude as the original growth rate (correction due to high-order interactions is not overwhelming). The production rates of resource or metabolites, $P_{i\alpha }$, are assumed to have mean $\mu_{P}/M$ and SD $\sigma_{P}/M$, which follows similar logic to $C_{i\alpha }$: total production of different resources of one consumer individual is of constant order. In addition to the parameter scaling introduced to ensure reasonable total rates when taking diversity to infinity, we can also make each prey abundance scale as 1/M by imagining a shared constant carrying capacity and then do not need to scale down consumption rates. Both methods lead to equivalent dynamics because we can scale prey abundances in the latter case by M which automatically cause consumption rates to scale by 1/M in the equations.

The local Jacobian can be obtained by standard linearization at the fixed point:

$$\begin{array}{c} J^{*}=\left[\begin{array}{cc} O & D(S^{*})(C⊙Y+R^{*}\cdot H+H\cdot R^{*})\\ P^{T}-D\left(R^{*}\right)C^{T} & \left[\frac{\partial h}{\partial R}\right]-D(C^{T}S^{*}) \end{array}\right], \end{array}$$

which is written as a block matrix if we concatenate predator vector and prey vector as [S;R] and consider its change around the fixed point. Here, we use $D(S^{*})$ for the diagonal matrix with vector $S^{*}$ on the diagonal, ⊙ to denote the Hadamard product, $R^{*}\cdot H$ to represent matrix $\sum_{\alpha }{R_{\alpha }^{*}H}_{i\alpha \beta }$, $H\cdot R^{*}$ for $\sum_{\beta }H_{i\alpha \beta }R_{\beta }^{*}$, and $\left[\frac{\partial h}{\partial R}\right]$ for the matrix with elements $\frac{\partial h_{\alpha }}{\partial R_{\beta }}$.

For different supply/growth functions, the lower right block (effective prey self-regulation) is given as follows. For chemostat supply, $h_{\alpha }\left(R_{\alpha }\right)=l_{\alpha }\left(\kappa_{\alpha }-R_{\alpha }\right)$ and therefore

$$\left[\frac{\partial h}{\partial R}\right]-D\left(C^{T}S^{*}\right)=-D\left(l\right)-D\left(C^{T}S^{*}\right).$$

And for logistic growth, $h_{\alpha }\left(R_{\alpha }\right)=g_{\alpha }R_{\alpha }\left(K_{\alpha }-R_{\alpha }\right)$, which gives

$$\left[\frac{\partial h}{\partial R}\right]-D\left(C^{T}S^{*}\right)=-D\left(g\right)D\left(R^{*}\right).$$

Note that in calculation, we have used the fixed-point property, $h_{\alpha }\left(R_{\alpha }^{*}\right)-$$R_{\alpha }^{*}\sum_{i}C_{i\alpha }S_{i}^{*}=0$. Regardless of different supply/growth functions, the lower right block is diagonal where all elements are negative (thus it represents self-regulation). Besides, each element in the diagonal is of the order of constant (not scaling with system sizes), which is important for later reasoning.

The stability of this Jacobian highly depends on the upper right block,

$$\Lambda :=D\left(S^{*}\right)\left(C⊙Y+R^{*}\cdot H+H\cdot R^{*}\right),$$

and the lower left one,

$$V^{T}:=P^{T}-D\left(R^{*}\right)C^{T}.$$

We consider an approximation where the lower right part, i.e., the effective self-regulation, is proportional to identity matrix, and rewrite the Jacobian as

$$J^{*}=\left[\begin{array}{cc} O & \Lambda \\ V^{T} & -a_{eff}I \end{array}\right].$$

Since the lower right part should be negative, we restrict $a_{eff}$ to be a positive number. Let $\lambda^{J}$ be the eigenvalue of the Jacobian, which can be solved from

$$\det\left(\lambda^{J}I-J^{*}\right)=0.$$

With the standard linear algebra technique for block matrix, when N<M, we can write the above equation as

$$\text{det}\left(\lambda^{J}I-\Lambda \frac{1}{\lambda^{J}+a_{\mathrm{eff}}}V^{T}\right)\text{det}(\lambda^{J}I+a_{\mathrm{eff}}I)=0,$$

which can be further simplified as

$$\text{det}\left(\lambda^{J}\left(\lambda^{J}+a_{\mathrm{eff}}\right)I-\Lambda V^{T}\right){\left(\lambda^{J}+a_{\mathrm{eff}}\right)}^{M-N}=0.$$

From this, we know there are M-N degenerate solutions $\lambda^{J}=-a_{eff}$, and another 2N solutions which can be obtained by solving

$$\lambda^{J}\left(\lambda^{J}+a_{\mathrm{eff}}\right)-\lambda_{i}^{\Lambda V^{T}}=0,\left(i=1,\cdots ,N\right),$$

where $\lambda_{i}^{\Lambda V^{T}}$ is the i th eigenvalue of $\Lambda V^{T}$. For cases when N>M, similarly, we can break down the original determinant equation into

$$\text{det}(\lambda^{J}I)\text{det}\left((\lambda^{J}+a_{\mathrm{eff}})I-V^{T}\frac{1}{\lambda^{J}}\Lambda \right)=0,$$

and then

$${\left(\lambda^{J}\right)}^{N-M}\text{det}\left(\lambda^{J}(\lambda^{J}+a_{\mathrm{eff}})I-V^{T}\Lambda \right)=0.$$

Besides the N-M degenerate solutions $\lambda^{J}=0$, we can have the rest 2M eigenvalues solved from

$$\lambda^{J}\left(\lambda^{J}+a_{\mathrm{eff}}\right)-\lambda_{\alpha }^{V^{T}\Lambda }=0,\;\left(\alpha =1,\cdots ,M\right).$$

Similarly, we use $\lambda_{\alpha }^{V^{T}\Lambda }$ to denote the α th eigenvalue of $V^{T}\Lambda$. To summarize, the positive eigenvalues of the Jacobian can be only obtained from the quadratic equations related to eigenvalues of $\Lambda V^{T}$ or $V^{T}\Lambda$.

Based on the scaling of different terms ($C_{i\alpha }$, $Y_{i\alpha }$, $S_{i}^{*}$, $R_{\alpha }^{*}$), we know that the eigenvalues of $\Lambda V^{T}$ or $V^{T}\Lambda$ have the magnitude 1/M or 1/N. This result is because $\Lambda V^{T}$ has the same scaling behavior as ${\mathrm{CC}}^{T}$ ($Y_{i\alpha }$, $S_{i}^{*}$, $R_{\alpha }^{*}$ are of constant order), whose eigenvalues are proved to scale as 1/M (54, 69, 70) (since SD of C element scales as 1/M). While the effective self-regulation is of constant order. In the limit of large N and large M, i.e., large complex system, $a_{eff}$ is much larger, and therefore, whether $\lambda^{J}$ can be positive is determined by whether the maximum (real part) $\lambda_{i}^{\Lambda V^{T}}$ or $\lambda_{\alpha }^{V^{T}\Lambda }$ is positive. For large complex systems, we reduce the stability problem to a problem of studying $\Lambda V^{T}$ or $V^{T}\Lambda$.

Note that although we can reduce the study of Jacobian to that of $\Lambda V^{T}$ or $V^{T}\Lambda$ via assuming that effective self-regulation matrix is proportional to an identity matrix, our numerical results show that the reduction to $\Lambda V^{T}$ or $V^{T}\Lambda$ is robustly correct when this assumption is not satisfied. A heuristic explanation is that the self-regulation terms can be all taken as infinity comparing to Λ and V in large communities, and thus are similar to being the same for Λ and V.

Next, we study the stability criterion for random matrix $\Lambda V^{T}$ or $V^{T}\Lambda$. If we have

$$\mathrm{Corr}\left(\Lambda_{i\alpha },V_{j\beta }\right)=-\rho \delta_{\mathrm{ij}}\delta_{\alpha \beta },$$

based on the random theory for nonsymmetric Wishart matrix, the non-Hermitian Marchenko–Pastur law (54, 69, 70), we can know $\Lambda V^{T}$ has positive eigenvalues when N≤M once

$$\rho \geq \sqrt{N/M},$$

and $V^{T}\Lambda$ has positive eigenvalues when N≥M if

$$\rho \geq \sqrt{M/N}.$$

Therefore, we can combine the two cases to obtain a compact stability criterion as

$$\rho \geq \sqrt{\text{min}\left(N/M,M/N\right)}.$$

Note that the original (non-Hermitian) Marchenko–Pastur law requires the matrices to be i.i.d. and mean zero. Here, both Λ and V matrices have nonzero mean. However, the nonzero mean can be regarded as a low rank perturbation to the zero-mean part (85), which would result in some negative outlier eigenvalues not affecting stability. Therefore, we can still apply the Marchenko–Pastur law here. Also, the presence of high-order regulation ($H_{i\alpha \beta }$) will make elements within Λ correlated, not satisfying above assumption of correlation between elements exactly. But this correlation within Λ decays with increasing M, and thus the Marchenko–Pastur law prediction can be accurate in large communities.

We now try to express the abstract correlation coefficient by mechanistic quantities, e.g., the variation in yields, the statistics of production rates, as well as the statistics of high-order regulation effects. We estimate the correlation ρ for the equal concentration and equal abundance cases as previous works (41, 86, 87), which is shown to work for general cases by numerical experiments. The SD of Λ matrix element is approximated by

$$\sigma_{\Lambda }=\frac{\mu_{S}}{M}\sqrt{\sigma_{C}^{2}\sigma_{Y}^{2}+\sigma_{C}^{2}\mu_{Y}^{2}+\sigma_{Y}^{2}\mu_{C}^{2}+2\mu_{R}^{2}\sigma_{H}^{2}}.$$

The SD of V matrix element is approximated as

$$\sigma_{V}=\frac{1}{M}\sqrt{\sigma_{P}^{2}+\mu_{R}^{2}\sigma_{C}^{2}}.$$

The covariance between $\Lambda_{i\alpha }$ and $V_{i\alpha }$ is

$$\mathrm{Cov}\left(\Lambda_{i\alpha },V_{i\alpha }\right)=-\mu_{R}\mu_{S}\sigma_{C}^{2}\mu_{Y}/M^{2}.$$

At the end, we have the estimated correlation as

$$\rho_{\mathrm{est}}=\frac{-\mathrm{Cov}\left(\Lambda_{i\alpha },V_{i\alpha }\right)}{\sigma_{\Lambda }\sigma_{V}}=\sqrt{\frac{1}{\left(1+\frac{\sigma_{P}^{2}}{\mu_{R}^{2}\sigma_{C}^{2}}\right)\left(1+\frac{\sigma_{Y}^{2}}{\mu_{Y}^{2}}+\frac{\sigma_{Y}^{2}\mu_{C}^{2}}{\sigma_{C}^{2}\mu_{Y}^{2}}+\frac{2\mu_{R}^{2}\sigma_{H}^{2}}{\sigma_{C}^{2}\mu_{Y}^{2}}\right).}}$$

Substituting the estimated correlation into the stability criterion, we can obtain the two possible regions of diversity permitting stability,

$$\begin{array}{c} N\leq \frac{M}{\left(1+\frac{\sigma_{P}^{2}}{\mu_{R}^{2}\sigma_{C}^{2}}\right)\left(1+\frac{\sigma_{Y}^{2}}{\mu_{Y}^{2}}+\frac{\sigma_{Y}^{2}\mu_{C}^{2}}{\sigma_{C}^{2}\mu_{Y}^{2}}+\frac{2\mu_{R}^{2}\sigma_{H}^{2}}{\sigma_{C}^{2}\mu_{Y}^{2}}\right)},\left(N\leq M\right), \end{array}$$

$$\begin{array}{c} N\geq M\left(1+\frac{\sigma_{P}^{2}}{\mu_{R}^{2}\sigma_{C}^{2}}\right)\left(1+\frac{\sigma_{Y}^{2}}{\mu_{Y}^{2}}+\frac{\sigma_{Y}^{2}\mu_{C}^{2}}{\sigma_{C}^{2}\mu_{Y}^{2}}+\frac{2\mu_{R}^{2}\sigma_{H}^{2}}{\sigma_{C}^{2}\mu_{Y}^{2}}\right),\left(N\geq M\right). \end{array}$$

Heuristically comparing to May’s result (possible in the regime N≤M) (28), where we have $\sigma \sqrt{N}\leq 1$ as the stability criterion, we can express the phenomenological interaction strength by the mechanistic variables as

$$\sigma =\sqrt{\frac{1}{M}\left(1+\frac{\sigma_{P}^{2}}{\mu_{R}^{2}\sigma_{C}^{2}}\right)\left(1+\frac{\sigma_{Y}^{2}}{\mu_{Y}^{2}}+\frac{\sigma_{Y}^{2}\mu_{C}^{2}}{\sigma_{C}^{2}\mu_{Y}^{2}}+\frac{2\mu_{R}^{2}\sigma_{H}^{2}}{\sigma_{C}^{2}\mu_{Y}^{2}}\right)}.$$

We use Julia to conduct numerical tests and run simulations and use Python for data analysis and drawing figures. Simulations are run on MIT Supercloud (88). All codes are available at https://github.com/liuyz0/Critical-match (89). For a simulation process, we first determine the community diversities, i.e., the number of predators and that of prey. We then sample the parameters like consumption and growth rates based on given statistical parameters like mean and variance. We next sample the fixed point with given statistical parameters like mean and variance. Other parameters as mortalities and those in the prey/resource growth/supply function can be solved from the fixed point. We next can simulate the ordinary differential equations with various initial conditions. Time series outputted can give the information about survival diversity and fluctuating or not. After collecting all final states from different initial conditions, if the final states are all stable, we can use principal component analysis to tell whether they are the same states (globally stable state) or not (alternative stable states). Except Fig. 1, which uses simulation data, we mainly did numerical experiments focusing on numerical analysis of the Jacobian without simulating the full dynamics. The sampling process is the same while stability can be directly read out from the eigenvalues of the numerically calculated local Jacobian eigenvalues.

In sampling yields, we used the Gaussian distribution given mean, $\mu_{Y}$, and SD, $\sigma_{Y}$. We may encounter yield values greater than 1, which seems to violate mass conservation. In general, the yield values can be arbitrarily large if we use different units for prey and predator abundances. If we are forced to use the same unit for all abundances, the yields should then be smaller than 1. In this case, we can multiply a small number to yields after sampling from the Gaussian distribution to make sure the yield values are below 1. Note that both mean and SD will be scaled by the same factor, the yield variation remains the same as $\sigma_{Y}/\mu_{Y}$. Therefore, our results are not affected by restricting values of yields to be smaller than 1, essentially because stability does not depend on magnitudes but dimensionless ratios.

## Supplementary Material

Appendix 01 (PDF)

## Data Availability

Code and simulation data have been deposited in https://github.com/liuyz0/Critical-match (89).
